# Unveiling the Ultralow Thermal Conductivity of Hybrid Perovskites

**DOI:** 10.1002/advs.76841

**Published:** 2026-08-05

**Authors:** Qinqin He, Yuli Chen, Zhigang Li, Yanguang Zhou

**Affiliations:** ^1^ Department of Mechanical and Aerospace Engineering The Hong Kong University of Science and Technology Kowloon Hong Kong SAR People's Republic of China; ^2^ Institute of Solid Mechanics Beihang University Beijing People's Republic of China; ^3^ Collaborative HKUST Shenzhen‐Hong Kong Collaborative Innovation Research Institute Futian Shenzhen Guangdong China

**Keywords:** chemical physics, lattice energy, materials science, octahedron, thermal conduction, thermal conductivity, thermal energy, thermal management of electronic devices and systems, thermal transport

## Abstract

Hybrid perovskites are among the most important photon‐to‐electricity materials, whose performance is strongly limited by poor heat dissipation. This is due to their significantly low thermal conductivity, and the origin of such low thermal conductivity is still in debate and much less well‐documented. In this paper, we experimentally observe that the thermal conductivity of hybrid perovskites is low and possesses a weak temperature dependence. We further show that this ultralow thermal conductivity of hybrid perovskites is caused by the disorder of organic molecules using atomistic simulations. This disorder strongly scatters the lattice vibrations and hinders the thermal transport in hybrid perovskites. Our calculations further show that the temperature dependence of the vibrational thermal conductivity results from the lattice framework transits from ∼*T* ^0^ to ∼*T* ^−1^, and the value doubles at room‐temperature and increases four‐fold at 150 K when the disorder introduced by organic molecules is excluded. Meanwhile, based on atomistic simulations, the thermal energy in hybrid perovskites is found to be transferred by vibrations of the lattice framework (∼48% to ∼65%) and the organic molecules (∼35% to ∼52%). We map heat transport through inorganic octahedral cages and organic molecules, clarify their mutual coupling and interference, and guide thermal management in perovskites.

## Graphical abstract

In hybrid perovskites, thermal energy flows through lattice vibrations of the framework (48%–65%) and organic molecules (35%–52%). When the disorder introduced by organic molecules is excluded, the temperature dependence of the vibrational thermal conductivity results from the lattice framework transits from ∼*T* ^0^ to ∼*T* ^−1^, and the value doubles at room‐temperature and increases four‐fold at 150 K.

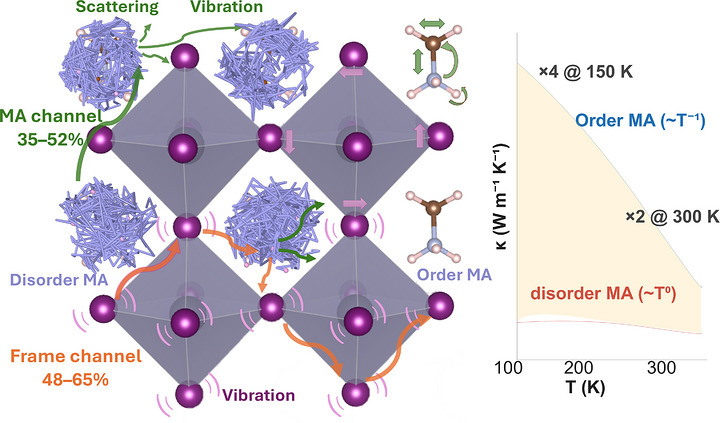

## 1 Introduction

The applications of hybrid perovskite‐based photovoltaics are limited by their ultralow thermal conductivity, i.e., usually lower than ∼1 W/mK at room‐temperature [1, 2, 3, 4, 5], which strongly hinders their heat dissipation capacity during operation. It is therefore necessary to increase the thermal conductivity of hybrid perovskites, which requires a comprehensive and accurate understanding of the thermal transport mechanism in them.

For example, Weadock et al. [6] determined the dynamic tilting of the octahedral framework in MAPbI_3_, which induces specific properties in ferroelectric domains, such as increasing the charge carrier lifetime. However, the detailed mechanism of thermal transport and the dynamics of heat carriers in such hybrid crystals are still in debate and much less well documented. Previous experiments [7, 8, 9, 10] and calculations [7, 8, 11, 12, 13, 14, 15] show that the thermal conductivity of MAPbI_3_ is 0.3–0.6 W/mK. Some researchers attribute this low thermal conductivity to the weak chemical bonds [7], which causes the low elastic stiffness and then the low sound velocity [16, 17]. Some researchers, on the other side, have plausibly ascribed this low thermal conductivity to the role of organic MA molecules, such as the vibrational resonant scatterings resulting from the confined diffusion of organic molecules [14], or the rotation of organic molecules [17, 18]. Furthermore, it is deduced that the rotation of organic molecules may provide an additional thermal transport channel in MAPbI_3_ [19]. These diverse explanations for the ultralow thermal conductivity of MAPbI_3_ raise a question: how do the organic molecules in MAPbI_3_ affect the corresponding thermal transport? Meanwhile, it has been found that the temperature‐dependent thermal conductivity of hybrid perovskites deviates from the classic *T* ^−1^ dependence in conventional crystals such as silicon and diamond [20, 21, 22].

In this paper, our atomistic simulations and experimental measurements demonstrate that the ultralow thermal conductivity of hybrid perovskites stems from the disorder caused by the motion of organic molecules. Instead of inferring how the structure influences the properties of perovskite [3, 7, 14], the connection between disorder and heat transfer in MAPbI_3_ has been directly quantified in this work. Our results show that the vibrational thermal conductivity of MAPbI_3_ follows the classic *T* ^−1^ relation when the disorder of organic molecules is excluded and becomes almost temperature‐independent if the disorder of organic molecules is considered. At 150 K, the vibrational thermal conductivity of hybrid perovskites increases by around four times when the disorder induced by organic molecules is excluded.

## 2 Results

### 2.1 Sample Preparation and Measurement

**FIGURE 1:**
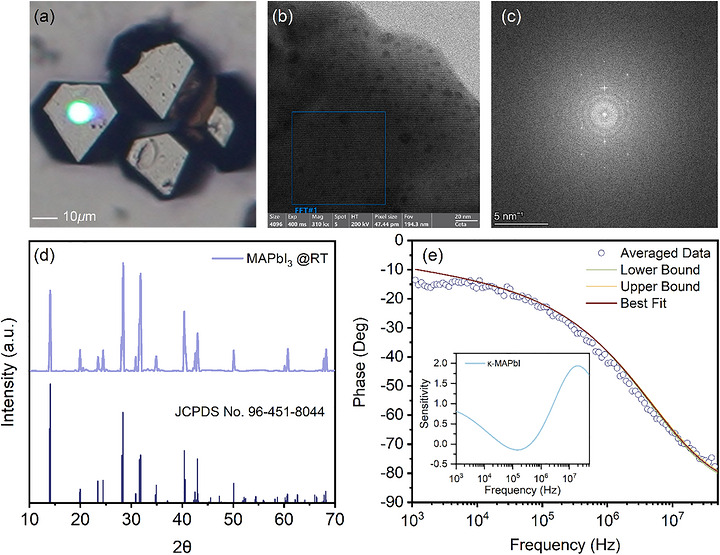
(a) The MAPbI_3_ sample together with the pump and probe laser during the test. (b) The HRTEM images of MAPbI_3_ pieces and (c) the corresponding fast Fourier transform spots show the single‐crystalline nature. (d) The PXRD patterns of the MAPbI_3_ and the comparison with JCPDS card No. 96‐451‐8044. (e) The phase lag between probe and pump laser (open blue circle) with the corresponding fitting curve (colorful solid lines). The inset picture shows the sensitivity of the thermal conductivity of the MAPbI_3_.

We first synthesize the single‐crystalline MAPbI_3_ and measure the corresponding thermal conductivity at 140–380 K. The synthesis of single‐crystalline MAPbI_3_ (Figure 1a) follows procedures reported by our colleague Wang et al. [9]. The high‐resolution transmission electron microscopy (HRTEM) images and the corresponding fast Fourier transform (FFT) patterns (Figure 1b,c) confirm the purity and crystallinity of our samples. This is further confirmed by the powder X‐ray diffraction patterns (PXRD), which agree well with JCPDS card No. 96‐451‐8044 and previous reports [9, 23] (Figure 1d). The synthesis and characterization details are given in the Methods.

The thermal conductivity of single‐crystal MAPbI_3_ is then measured using optical pump‐probe spectroscopy based on the frequency domain thermoreflectance (FDTR, see Methods for details). In our FDTR measurements, Au served as the transducer and was heated by the pump laser during the FDTR measurements (see Note S1 for details). Here, high‐quality Au/MAPbI_3_ heterointerfaces with flat and smooth surfaces (see Note S2 for details) were chosen in the FDTR measurements to ensure a good thermoreflectance response. Generally, the sample with parallel surfaces and uniform transducer layers can facilitate our measurements. This can be confirmed by the laser profiles obtained through the optical microscope (Figure 1a). Then, the phase lag between the pump beam and the probe beam measured by a lock‐in amplifier in our FDTR measurements was fitted using a heat diffusion model [24, 25, 26, 27]. The thermal conductivity (κ) of MAPbI_3_ can then be determined. In the FDTR measurements, the intensity radius for the probe and the pump laser beams were 5 and 3.76 *µ*m, respectively. The measured data were collected under a ten times optical microscope. Figure 1e shows one representative FDTR measurement at room‐temperature. The low temperatures, i.e., 140–290 K, in our measurements are controlled using a chamber with liquid nitrogen. The chamber was sealed and evacuated with a vacuum pump to eliminate the influence of moisture. The high temperatures, i.e., 290–380 K, are achieved using a temperature‐controlled stage. The measured thermal conductivity is fitted based on the mean value with six samples and at least three sweeps per sample (seeNote S2 for details).

### 2.2 Thermal Conductivity Calculations

**FIGURE 2:**
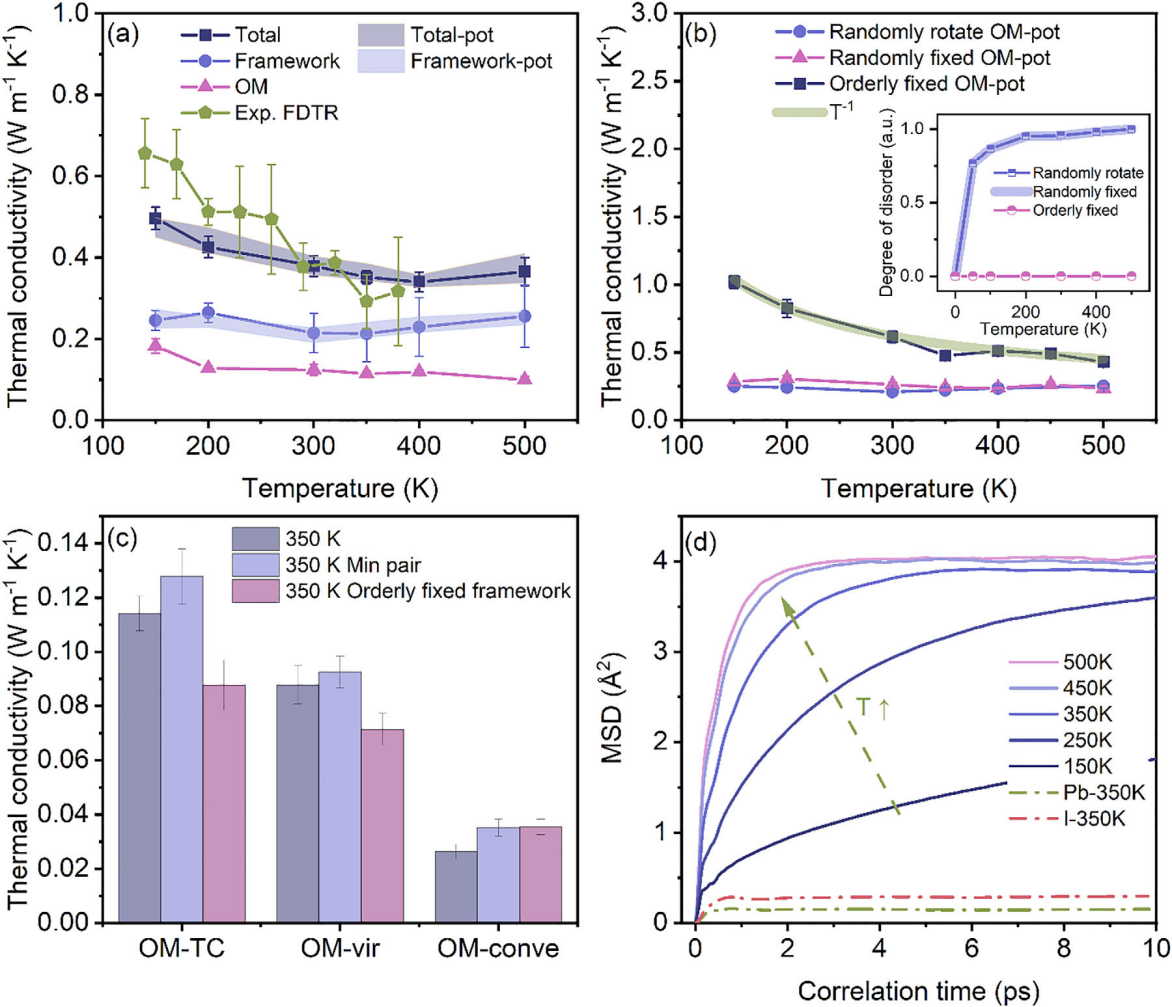
(a) The temperature‐dependent thermal conductivity of the hybrid perovskite MAPbI_3_, considering contributions from organic molecules and framework. (b) The lattice thermal conductivity of the framework of MAPbI_3_ with random, randomly fixed, and orderly fixed organic molecules. Here, the lattice thermal conductivity of the framework is approximated by the potential heat flux Equations (2) and (3). The inset plot represents the degree of disorder(DOD) for hybrid perovskite MAPbI_3_ with random, randomly fixed, and orderly fixed organic molecules at various temperatures. (c) The thermal conductivity contributed by organic molecules at 350 K with/without special treatments by freezing the framework and minimizing the interaction (i.e., 50% of the original value) between organic molecules and the cage. (d) The MSD of organic molecules at various temperatures ranging from 150 to 500 K. The dotted‐dashed lines are the MSD of atoms in the framework at 350 K.

The thermal conductivity of hybrid perovskites is calculated based on the linear response theory through the Green‐Kubo formula [28, 29, 30]

(1) $$\kappa =\frac{V}{3k_{b}T^{2}}\int_{0}^{\infty }\langle {\vec{Q}}_{total}\left(\tau \right){\vec{Q}}_{total}\left(0\right)\rangle d\tau$$

where *T* is the system temperature, *V* is the system volume, and *k_b_* is the Boltzmann constant. ${\vec{Q}}_{total}$ denotes the total heat flux, and is calculated using

(2) $${\vec{Q}}_{total}={\vec{Q}}^{kin}+{\vec{Q}}^{pot}=\frac{1}{V}\sum_{i}E_{i}{\vec{v}}_{i}+\frac{1}{V}\sum_{i,j\neq i}{\vec{v}}_{i}\cdot \left(\frac{\partial U_{j}}{\partial {\vec{r}}_{ji}}⊗{\vec{r}}_{ij}\right)$$

in which, *E_i_* is the atomic energy, including both potential and kinetic energy, ${\vec{v}}_{i}$ is the atomic velocity, *U_j_* is the potential energy, ${\vec{r}}_{ij}$ is the relative distance between atoms *i* and *j*. The computational details are provided in Note S3, where the thermal conductivity contributed by the potential heat flux and the total heat flux have been calculated over the temperature range of 150–500 K. Our results show that the thermal conductivity contributed from the kinetic heat flux is negligible, and the total thermal conductivity can be regarded as the contribution of potential heat flux (Figure 2a and Note S3). The potential heat flux includes both the contributions from the framework and organic molecules, i.e., 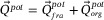. Then, the total thermal conductivity can be further decomposed into

(3) $$\begin{array}{cc} & \kappa \approx \frac{V}{3k_{b}T^{2}}\int_{0}^{\infty }\langle {\vec{Q}}^{pot}\left(\tau \right){\vec{Q}}^{pot}\left(0\right)\rangle d\tau \\ & =\underset{framework}{\underset{︸}{\frac{V}{3k_{b}T^{2}}\;\int_{0}^{\infty }\langle {\vec{Q}}_{fra}^{pot}\left(\tau \right){\vec{Q}}^{pot}\left(0\right)\rangle d\tau }}+\underset{organic\;molecules}{\underset{︸}{\frac{V}{3k_{b}T^{2}}\int_{0}^{\infty }\langle \;{\vec{Q}}_{org}^{pot}\left(\tau \right){\vec{Q}}^{pot}\left(0\right)\rangle d\tau }}\\ & =\kappa_{fra}+\kappa_{org} \end{array}$$

The heat current used to calculate the corresponding thermal conductivity is obtained by running a molecular dynamics (MD) simulation using the Large‐scale Atomic/Molecular Massively Parallel Simulator (LAMMPS) package [31]. The calculation details can be found in the Methods. Our calculated thermal conductivity of MAPbI_3_ is 0.38 ± 0.025 W/mK at room‐temperature and shows a weak temperature dependence, which agrees with our measured value (Figure 2a).

We then decompose the thermal conductivity into two parts, contributed from the framework and the organic molecules Equation (3). It is found that the vibrational thermal conductivity contributed by the framework, i.e., κ_*fra*_, shows a glass‐like behavior and is almost temperature‐independent (Figure 2a). Theκ_*fra*_ becomes 0.26 ± 0.018 W/mK at room‐temperature and remains constant at various temperatures when all organic molecules are randomly fixed (Figure 2b and see Note S3 for details). The κ_*fra*_ becomes 0.62 ± 0.042 W/mK at room‐temperature and strictly follows the classic *T* ^−1^ relationship when all organic molecules are orderly fixed (Figure 2b and see Note S4 for details). To understand this phenomenon, we propose a degree of disorder (DOD) to quantitatively characterize the disorder induced by the large diffusion of organic molecules (see Methods for details). Our calculated results show that the DOD for MAPbI_3_ with orderly fixed organic molecules is always zero (Figure 2b), while the DOD for MAPbI_3_ with random or randomly fixed organic molecules converges quickly with temperature. Even at low temperatures, the DOD for MAPbI_3_ with randomly or randomly fixed organic molecules is large, i.e., ∼0.8 at 50 K, which indicates organic molecules in systems are quite disordered. When the temperature increases to 200 K, organic molecules become fully disordered, and the corresponding DOD converges to unity. Therefore, the glass‐like κ_*fra*_ of hybrid crystals with randomly rotated or randomly fixed organic molecules stems from the disorder introduced by organic molecules.

We also calculate the autocorrelation function (ACF) of the rotation of the C─N bond, which indicates the relative change of the rotation of the C─N bond (see Note S5 for details). Our results show that the ACF decreases quickly from unity to small values when the temperature is over 200 K. This means the C─N bond rotates largely at temperatures over 200 K (Figure S10). When the temperature is lower than 200 K (Figure S10), the ACF stabilizes around a high value approaching unity, which indicates the C─N bond rotates a little compared to its initial position. The calculated temperature‐dependent ACF further confirms the validity of our defined DOD.

Apart from the simulation results, the disorder of organic molecules has also been experimentally characterized using quasi‐elastic neutron scattering (QENS) [32] and backscattering neutron spectroscopy [33]. In these experimental observations, two distinct reorientation modes of the organic molecules have been resolved with the CH_3_/NH_3_ rotation along the C─N axis (i.e., mode 1) and the corresponding tumbling of the C─N axis (i.e., mode 2). It has been found that the residence time of these rotational motions decreases with increasing temperature, i.e., the residence time of mode 1 decreases from 8 ps at 140 K to 2.6 ps at 370 K [32], which demonstrates that the disorder of organic molecules increases with the rise of temperature. The Raman spectrum of MAPbI_3_ further demonstrates that the disorder of organic molecules increases with the rise of temperature. In the Raman spectrum, two specific modes relating to organic molecules, i.e., the Raman shift of 315 cm^−1^ corresponding to the C─N torsion mode and the Raman shift of 968 cm^−1^ representing the C─N stretch mode, disappear when the temperature increases from 100 to 300 K [34]. The defined DOD captures the C─N orientational misalignment with respect to its original reference, which quantitatively characterizes the angular deviation from its ground‐state orientation. The change of DOD of MAPbI_3_ at various temperatures is therefore consistent with the experimental observations [32]. At the same time, our FDTR measurements show that the experimental thermal conductivity decreases from 0.66 ± 0.084 Wm^−1^K^−1^ at 140 K and 0.32 ± 0.13 Wm^−1^K^−1^ at 380 K. As shown in our simulations and previous results [32], the increase in disorder of organic molecules strengthens the scattering among heat carriers. We therefore deduce that the increase in the disorder of organic molecules decreases the corresponding thermal conductivity. Moreover, it has been demonstrated that organic molecules can be locked into their lowest‐energy orientations by the strong dipole–dipole interactions and cage‐deformation energy [32], which therefore provides the possibility to tune the thermal conductivity of hybrid perovskites using the disorder of organic molecules.

Meanwhile, organic molecules in hybrid crystals are found to provide an extra thermal pathway to transfer thermal energy, and the corresponding thermal conductivity, i.e., κ_*org*_, decreases with temperature (Figure 2a). We first reduce the interaction between the framework and organic molecules and find that the thermal conductivity contributed by organic molecules almost does not change (Figure 2c). This indicates that the thermal conductivity contributed by organic molecules stems from interactions between organic molecules rather than the mutual interaction between organic molecules and the framework. As mentioned above, organic molecules can largely diffuse inside the octahedral cage. Therefore, the thermal energy may be transferred by organic molecules through both their vibrations and collisions between them. However, the diffusion of organic molecules is confined in the octahedral cage, and the mean square displacement (MSD) converges when the temperature is higher than 350 K (Figure 2d). The trajectories of organic molecules in MAPbI_3_ show that the nearest distance between the trajectories of two adjacent organic molecules is 2.5 Å (Figure S7). This distance is almost beyond the interaction range used in our interatomic potential. Therefore, while organic molecules can largely diffuse inside the octahedral cage, the thermal energy transferred by organic molecules is solely through their vibrations. We also calculate the conductive thermal conductivity based on our recently proposed Onsager framework [35], where the conduction heat flux is calculated using

(4) $${\vec{Q}}_{cond}=\underset{total}{\underset{︸}{\frac{1}{V}\left[\sum_{i}E_{i}{\vec{v}}_{i}+\frac{1}{V}\sum_{i,j\neq i}{\vec{v}}_{i}\cdot \left(\frac{\partial U_{j}}{\partial {\vec{r}}_{ji}}⊗{\vec{r}}_{ij}\right)\right]}}-\underset{mass}{\underset{︸}{\frac{1}{V}\sum_{\alpha =1}^{2}h_{\alpha }\sum_{i=1}^{N_{\alpha }}{\vec{v}}_{\alpha i}}}$$

where the first two terms stand for the total heat flux and the second term represents the heat flux stemming from the mass transport, *E_i_* is the atomic energy, including both potential and kinetic energy, ${\vec{v}}_{i}$ is the atomic velocity, *U_j_* is the potential energy, ${\vec{r}}_{ij}$ is the relative distance between the atom *i* and *j*. *N*_α_is the number of particles for species *α*, and *h*_α_ denotes the average partial enthalpy of species *α* and can be calculated through

(5) $$h_{\alpha }=\frac{\sum_{i=1}^{N_{\alpha }}\left[K_{i}+P_{i}+\frac{1}{3}\left(m_{i}{\vec{v}}_{i}^{2}+\frac{1}{2}\sum_{j=1}^{N}{\vec{r}}_{ji}\cdot \frac{\partial U_{j}}{\partial {\vec{r}}_{ji}}\right)\right]}{N_{\alpha }}$$

in which, *K_i_* and *P_i_* are the time‐average kinetic and potential energies of particles of species *α*, respectively. Our results show that the thermal conductivity calculated using conductive, virial, and total heat flux is almost identical, which further confirms that the thermal transport channel stemming from the collision of organic molecules in MAPbI_3_ is negligible, as shown in Figure S7.

Here, we would like to emphasize that even the static disorder of organic molecules can lead to an almost temperature‐independent thermal conductivity for the framework of MAPbI_3_ (Figure 2b). Meanwhile, organic molecules can transfer thermal energy through proper wavelength vibrations. Therefore, the overall thermal conductivity of MAPbI_3_ shows a weak temperature dependence (Figure 2a). This may be important for investigating the thermal transport properties of MAPbI_3_ at extremely low temperatures, in which organic molecules move slowly while still generating disorder.

### 2.3 Spectral Analysis and Vibrational Lifetime

**FIGURE 3:**
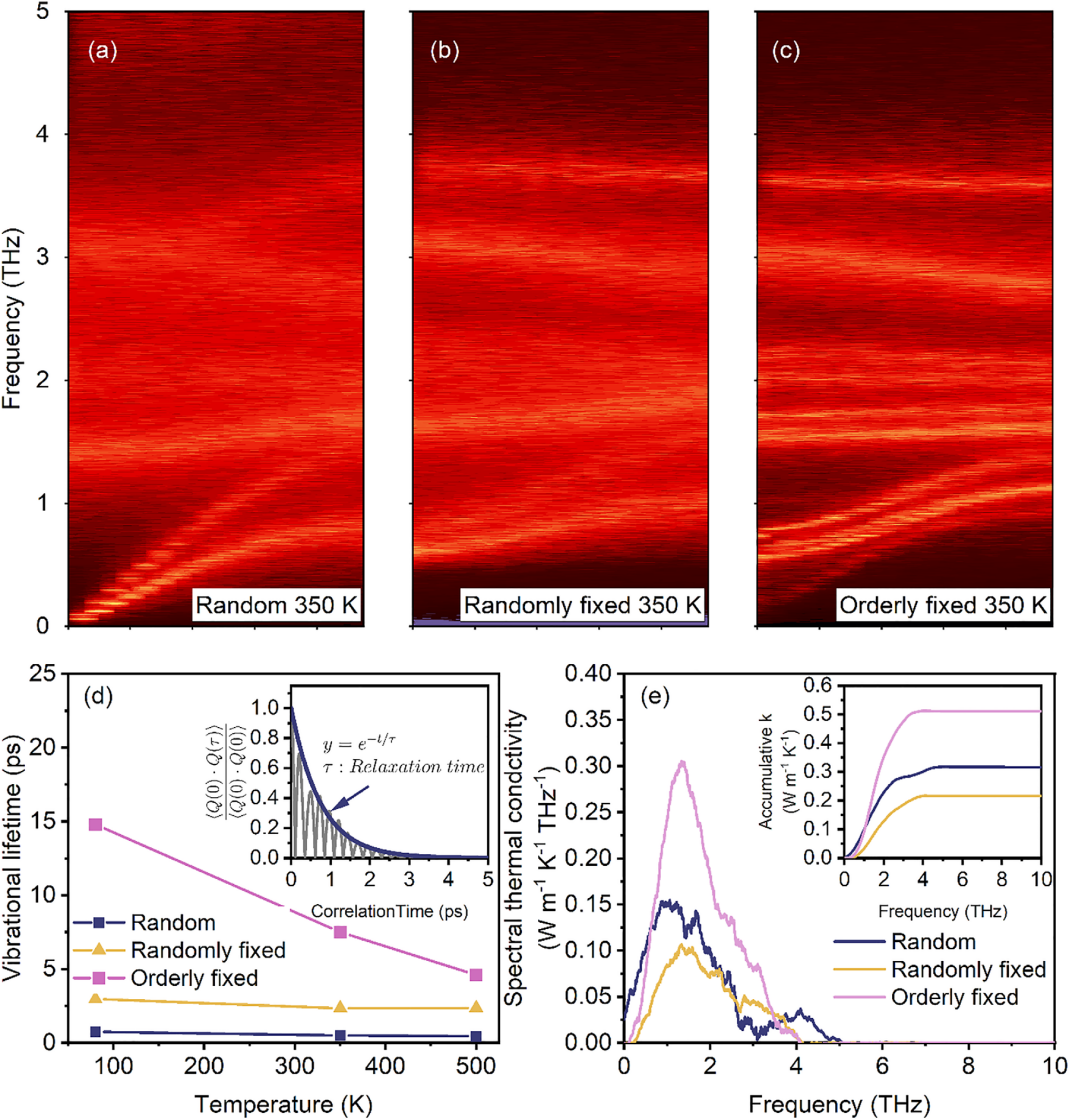
The spectral energy density of hybrid perovskite MAPbI_3_ with (a) random, (b) randomly fixed, and (c) orderly fixed organic molecules. (d) The vibrational lifetime of the corresponding systems was calculated by the exponential decay fitting of the heat flux. The inset shows the decay of the autocorrelation of the heat flux, which is used to calculate the vibrational lifetime [39]. (e) Spectral thermal conductivity and the accumulative thermal conductivity(inset) of MAPbI_3_ with random, randomly fixed, and orderly fixed organic molecules.

We next calculate the spectral energy densities (SEDs) [36, 37, 38] to qualitatively characterize the scattering picture in MAPbI_3_ with random, randomly fixed, and orderly fixed organic molecules (see Methods for details). Our results show that the linewidth in the SED of MAPbI_3_ with random organic molecules is similar to that of MAPbI_3_ with randomly fixed organic molecules (Figure 3a,b). The similar linewidth indicates that the strength of scattering among vibrations in these two systems is similar, which agrees well with our calculated thermal conductivities. i.e., the vibrational thermal conductivity of MAPbI_3_ is comparable to that of the MAPbI_3_ with randomly fixed organic molecules. However, the SED of MAPbI_3_ with orderly fixed organic molecules shows clear dispersions with small linewidths, indicating moderate scatterings among vibrations in the framework (Figure 3c). As a result, the thermal conductivity of MAPbI_3_ with orderly fixed organic molecules is much higher than that of MAPbI_3_ with random or randomly fixed organic molecules at a specific temperature (Figure 2b).

We further calculate the mean relaxation time of vibrations in MAPbI_3_ using $\tau =\int_{0}^{\infty }\frac{\langle {\vec{Q}}_{fra}^{pot}\left(\tau \right){\vec{Q}}^{pot}\left(0\right)\rangle }{\langle {\vec{Q}}_{fra}^{pot}\left(0\right){\vec{Q}}^{pot}\left(0\right)\rangle }d\tau$ [39] (Figure 3d). The estimated average relaxation time of vibrations in MAPbI_3_ with random or randomly fixed organic molecules is almost independent of temperature (Figure 3d), which again demonstrates that vibrations in these systems are significantly scattered by random organic molecules. On the contrary, the average relaxation time of vibrations in MAPbI_3_ with orderly fixed organic molecules decreases with the increase of temperature (Figure 3d). This is because organic molecules in the system are orderly fixed and scattering among these vibrations are dominated by Umklapp processes. As a result, the thermal conductivity of MAPbI_3_ with orderly fixed organic molecules shows a classic *T* ^−1^ relation as shown above (Figure 2b). It is known that the frequency of acoustic phonon branches at Gamma is exactly zero because it represents a uniform and rigid translation of the entire crystal lattice. When organic molecules are fixed, the whole crystal lattice cannot translate uniformly. Therefore, the frequency of the acoustic phonon branches at the Gamma points shifts up a little. However, we would like to emphasize that our systems with fixed organic molecules are still stable, as demonstrated by corresponding SEDs.

Furthermore, our spectral analysis shows that the thermal conductivity of all three cases is mainly contributed by vibrations with frequencies smaller than 6 THz. The calculation details can be found in Methods. When the organic molecules rotate randomly, the acoustic vibrations (i.e., their vibrational frequencies are smaller than 2 THz) contribute >50% to the total thermal conductivity (Figure 3e). The accumulative thermal conductivity of systems with randomly rotated OM, randomly fixed OM, and orderly fixed OM is 0.32, 0.21, and 0.51 W m^−1^K^−1^, respectively. In our EMD simulation, the thermal conductivity of the framework of these three systems is 0.38, 0.26, and 0.62 W m^−1^K^−1^, respectively. The difference between the NEMD results and the EMD results may be attributed to the NEMD simulation model not being long enough to eliminate size effects. We emphasize that the main purpose of the NEMD simulation is to decompose the thermal transport into the frequency domain, aiming to identify differences among the three systems rather than to calculate thermal conductivity. The spectral thermal conductivity generally decreases when the organic molecules are disordered, confirming that the introduction of disordered organic molecules suppresses the heat conduction.

### 2.4 X‐Ray Scattering and Neutron Scattering

**FIGURE 4:**
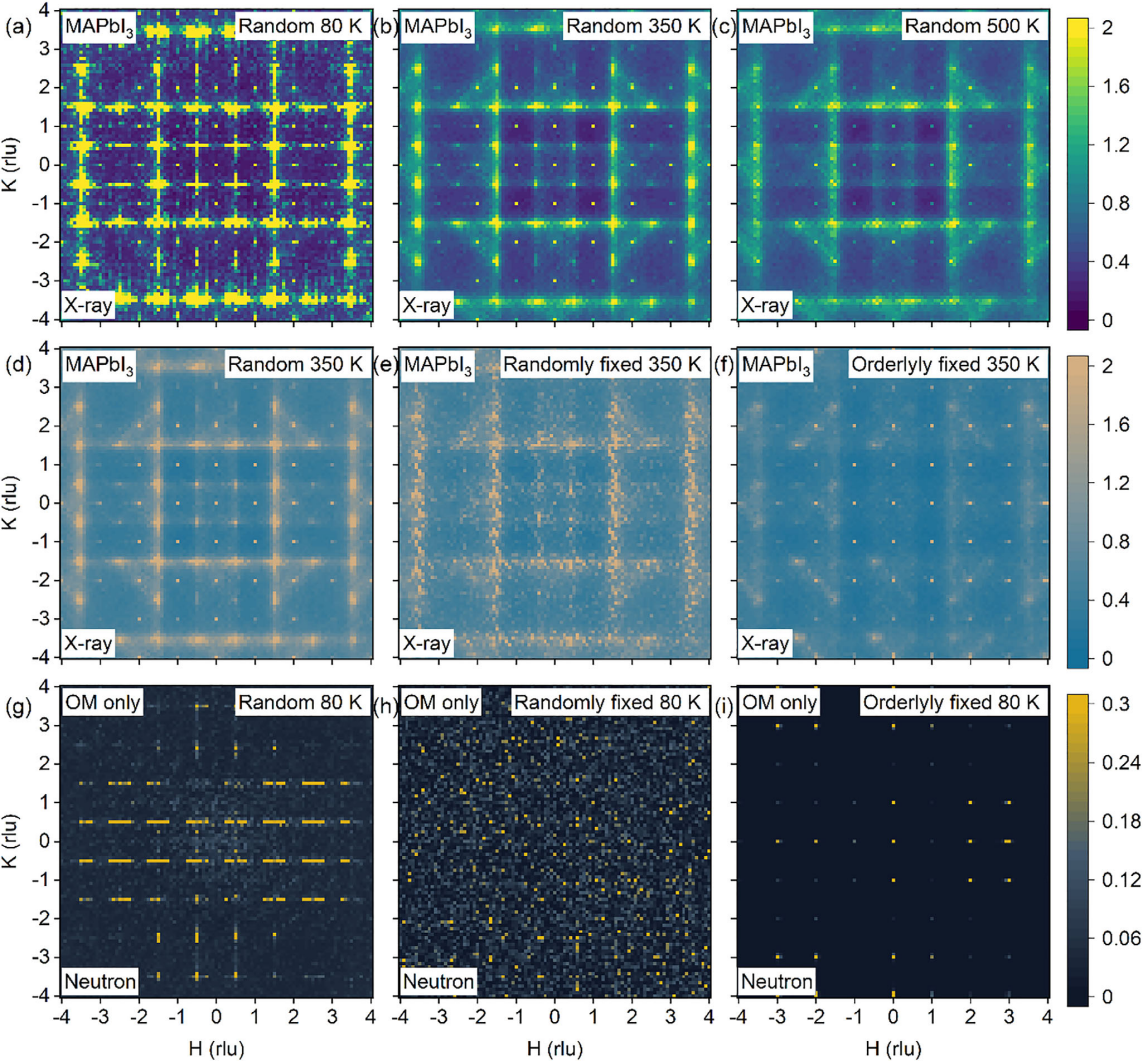
(a–c) The X‐ray scattering spectra for hybrid perovskite MAPbI_3_ at 80, 350, and 500 K, respectively. (d–f) The X‐ray of MAPbI_3_ with random, randomly fixed, and orderly fixed organic molecules at 350 K. (g–i) The neutron scattering spectra of random, randomly fixed, and orderly fixed organic molecules at 80 K. The rlu stands for reciprocal lattice unit.

It is known that X‐ray scattering and neutron scattering (NS) are widely applied to quantify the crystal structures containing heavy atoms [6, 40] and light atoms [6, 41], respectively. We first calculate the X‐ray scattering spectra of MAPbI_3_ at three typical temperatures, i.e., 80, 350, and 500 K (Figure 4). The calculation details can be found in the Methods. It is known that more phonons are occupied at higher temperatures. The total scattering signal, obtained by energy integration of the X‐ray or neutron scattering spectra, can be used to investigate the evolution of the atomic structure and its thermal disorder at various temperatures [6]. Our results show that the X‐ray scattering spectra show apparent sharp Bragg peaks and diffuse rods at the Brillouin zone at all temperatures considered here, and the corresponding intensity generally decreases with the increase of temperature (Figure 4a–c and see SI Note S6 for details). This is because the amplitude of atomic vibrations in the framework is small at low temperatures, leading to a more ordered atomic framework. It is noted that the X‐ray scattering spectra are not sensitive to light atoms, i.e., the organic molecules in hybrid perovskites. Therefore, the lattice structure of organic molecules cannot be clearly distinguished from the X‐ray scattering spectra.

We also calculate the X‐ray spectra for MAPbI_3_ with random, randomly fixed, and orderly fixed organic molecules at 350 K (Figure 4d–f). Our calculated diffuse scattering spectrum shows that the intensity of diffuse rods becomes much weaker when the organic molecules are orderly fixed, compared to those of systems with random and randomly fixed organic molecules (Figure 4d–i). Therefore, the diffuse rods in the diffuse scattering spectrum are caused by both the octahedral tilting and the disorder of organic molecules. Meanwhile, the background intensity in the diffuse scattering spectrum (Figures S13 and S14) can be used to reflect the inelastic scattering stemming from the disorder of organic molecules. The intensity of these dots away from the rods increases with the rise in temperature. This is due to the strong inelastic scattering caused by thermally excited lattice vibrations in the framework (Figure 4 and see Note S6 for details). The background intensity in the diffuse scattering spectrum for the system with the orderly fixed organic molecules is smaller than that in the other two cases (Figure S14). This suggests that a stronger inelastic X‑ray scattering caused by thermally excited lattice vibrations exists in MAPbI_3_ with random or randomly fixed organic molecules.

We further calculate the NS spectra for organic molecules in MAPbI_3_ with random, randomly fixed, and orderly fixed organic molecules at 80 K (Figure 4g–i). Here, we calculate the NS spectra of organic molecules rather than the whole system at 80 K. This is because the neutrons interact with the nucleus, and the yield NS intensity is from both the framework and the organic molecules [6, 42, 43]. The NS plotting, considering only organic molecules, clearly shows the behavior of the organic molecules. In contrast, the X‐ray interacts with the electronic cloud of atoms and therefore is sensitive to the mass of atoms [6, 42, 43]. In our X‐ray scattering spectra, the intensity is dominantly contributed by the framework. It is found that sharp Bragg peaks are clearly observed in the NS spectra when organic molecules are orderly fixed (Figure 4i). Both sharp Bragg peaks and diffuse rods appear in the NS spectra for MAPbI_3_ (Figure 4g), which further proves the contribution of disordered organic molecules to the diffuse rods rather than merely octahedral tilting [6]. When organic molecules are randomly fixed, both sharp Bragg peaks and diffuse rods in the corresponding NS spectra disappear (Figure 4h). This is because the measured NS scattering is governed by the dynamic structure factor $S(\vec{k},E)$, which is the space‐time Fourier transform of the density‐density correlation. The $S(\vec{k})$ spectra correspond to the energy‐integrated quantity $\int S(\vec{k},E)d\omega$ with probe‐specific weighting Equation (9). Once organic molecules are randomly fixed, the coherent scattering contribution becomes featureless as the random orientations suppress any periodic or correlated modulation, leaving a featureless diffuse background. This implies that organic molecules transfer thermal energy through vibrations, which again demonstrates our analysis above.

It is noted that Weadock et al. [6] has reconstructed the structure of hybrid perovskites in the Brillouin zone based on Neutron and X‐ray diffuse scattering and inelastic scattering, which unveils the mechanism of structure correlation between the dynamic octahedral sublattice and longer‐range intermolecular organic cations. While we have applied neutron and X‐ray diffuse scattering and inelastic scattering in our analysis, we focus on the underlying mechanism for the ultralow thermal conductivity of hybrid perovskites and their corresponding abnormal temperature dependence. Meanwhile, the background intensity of the X‐ray scattering (Figures S13 and S14) indicates that the strong inelastic scattering stems from the disorder of organic molecules. Our scattering spectrum analysis indicates that the diffuse rods in the neutral and X‐ray inelastic scattering spectrum are caused by both the octahedral tilting and disorder of organic molecules. Using equilibrium molecular dynamics simulations, we further demonstrate that the disorder induced by organic molecules, rather than the tilting of the dynamic octahedral sublattice, is the reason for the ultralow and weakly temperature‐dependent thermal conductivity of hybrid perovskites.

## 3 Conclusions

In conclusion, we have experimentally observed that the thermal conductivity of the single‐crystalline MAPbI_3_ is low and possesses a weak temperature dependence. Using atomistic simulations, we demonstrate that the ultralow thermal conductivity of hybrid perovskite MAPbI_3_ stems from the disorder caused by the confined diffusion of organic molecules. Our calculations further find that the thermal energy in MAPbI_3_ is transferred through both vibrations of the framework and organic molecules. The temperature dependence of the calculated vibrational thermal conductivity results from the framework changes from ∼*T* ^−0^ to ∼*T* ^−1^ when the state of organic molecules varies from randomly fixed or random to orderly fixed. This indicates vibrations in the framework are strongly scattered by disordered organic molecules. Meanwhile, organic molecules in hybrid crystals provide an extra thermal pathway for energy transfer through vibrations, with the corresponding thermal conductivity decreasing as temperature increases. Furthermore, our computed and existing measured spectral analysis unveils that the disorder induced by organic molecules significantly scatters vibrations and is the origin of the ultralow thermal conductivity of hybrid perovskites. Our study here unveils the microscopic picture of thermal energy transfer in hybrid perovskites, which facilitates the design of high‐performance hybrid perovskites.

## 4 Methods

### 4.1 Sample Preparation, Characterization, and Measurement

All the chemicals, including methylammonium iodide (MAI, 99.5%), PbI_2_ (99.9%, Aladdin), γ‐butyrolactone (GBL, 99%, Macklin), and poly (propylene glycol) (PPG‐3000, molecular weight 3000 Da, Aladdin), were used to synthesize MHHPs as received without further purification. For the synthesis of MAPbI_3_ single crystals, 2 mmol PbI_2_ and MAI were dissolved in 2 mL GBL and stirred at room‐temperature for 4 h, the solution was filtered using a PTFE filter with 0.2 µm pore size, the filtered solution was then sealed in a vial and kept in an oil bath at 95°C for crystallization. The details of the synthesis procedure can also be found in [9].

The powder XRD data of MAPbI_3_ were collected on the PANalytical Aeris powder‐X‐ray diffractometer with a Cu K_α1_/K_α2_ source (λ = 1.54051/1.54433 Å). The scanning transmission electron microscopy (STEM, JEM‐ARM200F JEOL) was then used to examine the lattice pattern of the synthesized crystalline MAPbI_3_. For STEM sample preparation, MAPbI_3_ powder was dispersed in diethyl ether and ultrasonicated to achieve a uniform suspension. Only the top layer of the liquid was extracted to drop on the Cu grid container, and this drop‐casting step was repeated three to five times to obtain films with sufficient thickness and particle loading for STEM imaging. High‐resolution TEM (HRTEM) images and the corresponding fast Fourier transform (FFT) patterns of MAPbI_3_ are therefore obtained. The specific heat capacity of MAPbI_3_ was measured by Differential Scanning Calorimeter tests (DSC 2500, TA) from 0°C to 100°C with a ramp rate of 3°C/min. The measured specific heat capacity is also compared with the calculated values using vibrational density of state as the input (see Note S2 for details).

The thermal conductivity of the synthesized single‐crystalline MAPbI_3_ was measured using frequency‐domain thermoreflectance (FDTR). This non‐contact technique measures the phase lag between the pump and probe beams, which arises from the samples’ thermal properties. A ∼100 nm gold layer was coated on the surface of the sample as the transducer to enhance the thermoreflectance signal, where gold has been demonstrated to have the highest coefficient of thermoreflectance [44]. The accurate thickness of the gold layer was further measured using the step meter, as the thickness will influence the thermal conductivity of the gold layer, and the thickness itself will also influence the fitting model. The thermal conductivity of the gold layer at various temperatures has been measured by FDTR (see Note S1 for details). FDTR measurements were performed with a 10× objective, which provides a radius of 3.6 µm for the pump laser and 5 µm for the probe laser. The laser spots were modelled with Gaussian profiles [24, 45]. Thermal transport properties were obtained by fitting the measured phase lag to a multilayer thermal diffusion model. The corresponding sensitivity analysis is given in Note S2.

### 4.2 Molecular Dynamics Simulations

In our work, the thermal conductivity of MAPbI_3_ is calculated using equilibrium molecular dynamics (EMD) simulations. All simulations were performed with the Large‐scale Atomic/Molecular Massively Parallel Simulator (LAMMPS) package [31]. The interatomic interactions are depicted by the MYP potential [46], which has been demonstrated to reproduce the thermal transport properties of MAPbI_3_ well. A 12 × 12 × 12 supercell, which is large enough to eliminate the size effect, is applied to implement MD simulations. The temperature ranges from 80 to 500 K. All models are first relaxed in an NPT ensemble (constant particle number, pressure, and temperature) for 50 ps with a timestep of 0.5 fs to eliminate the residual stress in the systems and then switched into an NVT ensemble (constant particle number, volume, and temperature) for another 50 ps. After that, simulations in an NVE (constant particle number, volume, and energy) are performed for another 0.75 ns, and the heat flux is output every eight steps for further calculation. Each calculated thermal conductivity is averaged using at least 12 independent simulations (see Note S4 for details).

The DOD is Defined as

(6) $$DOD\left(T\right)=\frac{\langle \sigma (T)\rangle -\min\langle \sigma (T)\rangle }{\max\langle \sigma (T)\rangle -\min\langle \sigma (T)\rangle }$$

where 〈σ(*T*)〉is the average standard deviation of σ^(*t*)^(*T*) at the temperature *T*, which is calculated via

(7) $$\sigma^{\left(t\right)}\left(T\right)=\sqrt{\frac{1}{M-1}\sum_{i=1}^{M}{\left(\theta_{i}^{\left(t\right)}\left(T\right)-\langle \theta^{\left(t\right)}\left(T\right)\rangle \right)}^{2}}$$

in which, $\theta_{i}^{\left(t\right)}\left(T\right)$ is the angle between the instantaneous C─N vector ${\vec{b}}_{i}^{\left(t\right)}\left(T\right)$ and the corresponding reference at *t* = 0, i.e., ${\vec{b}}_{i}^{\left(0\right)}\left(T\right)$, *M* is the number of C‐N vectors in the system, and $\left\langle \theta^{t}\left(T\right)\right\rangle =\frac{1}{M}\sum_{i=1}^{M}\theta_{i}^{\left(t\right)}\left(T\right)$. The DOD then captures the C─N orientational misalignment with respect to the reference, which quantitatively characterizes the angular deviation from its ground‐state orientation. Based on this definition, the DOD of a fully disordered and ordered system should be unity and zero, respectively. To validate the robustness of DOD metrics, the DOD of the organic molecules in MAPbBr_3_ and the O─H bond in water molecules have been calculated (see Note S5 for details).

The Spectral Energy Densities (SEDs) of the MAPbI_3_ with random, randomly fixed, and orderly fixed organic molecules are calculated through

(8) $$\begin{array}{cc} \Phi (\vec{k}) & =\frac{1}{4\pi \tau_{0}N}\sum_{\alpha }\sum_{b}^{B}m_{b}\left|\int_{0}^{\tau_{0}}\sum_{n_{x,y,z}}^{N}v_{\alpha }\left(\begin{array}{c} n_{x,y,z}\\ b \end{array};t\right)\right.\\ & \;{\left.\times \exp\left[i\vec{k}\cdot \vec{r}\left(\begin{array}{c} n_{x,y,z}\\ 0 \end{array}\right)-i\omega t\right]dt\right|}^{2} \end{array}$$

where τ_0_ is the integration time, which should be long enough, *N* is the total number of unit cells, *m_b_* is the mass of the basic atom *b* in the unit cell, ν_α_ is the atomic velocity along the α direction, *n*_*x*,*y*, *z*_ is the index number of unit cells along *x*, *y* and *z* directions [36]. The systems with a size of 8 × 8 × 40‐unit cells were run in an NVE ensemble for 2 million steps, in which the atomic position and velocity were recorded.

The X‐ray and NS spectra are calculated based on the dynamical structure factor, which can be obtained through

(9) $$S\left(\vec{k},E\right)={\left|\sum_{i}^{N}f_{i}\left(\vec{k}\right)\int \exp i\left[\vec{k}\cdot {\vec{r}}_{i}\left(t\right)-\frac{E}{\hbar }t\right]dt\right|}^{2}$$

where $\vec{k}$ is the momentum transfer of the scattered radiation, *E* = ℏω is the energy transferred into the material, and is summed over all *N* atoms [6]. For neutron scattering, $f_{i}\left(\vec{k}\right)\equiv b$ is the scattering length, which is independent of the momentum transfer. For X‐ray scattering, $f_{i}\left(\vec{k}\right)$ is the *k*‐dependent atomic form factor. The trajectories ${\vec{r}}_{i}\left(t\right)$ are sampled from MD simulations. The calculation was processed through the Python code developed by Sterling et al. [47]. The corresponding computational details are given in the Note S5. The elastic scattering of neutrons is characterized by sharp Bragg peaks at the center of the Brillouin zone for a well‐ordered crystal. These diffuse rods along the edges of the Brillouin zone indicate a correlation of the structure at a two‐dimensional (2D) plane. The spectra contributed from the framework (organic molecules) are obtained by setting the atomic form factor $f_{i}\left(\vec{k}\right)$ of organic molecules (the framework) to zero.

The spectral thermal conductivity can quantitatively characterize the thermal transport in MAPbI_3_. We calculate the spectral thermal conductivity of MAPbI_3_ with random, randomly fixed, and orderly fixed organic molecules (Figure 3e) by utilizing the frequency‐domain direct decomposed method (FDDDM) [48, 49, 50]. For NEMD simulations, the size of the system is 50 × 50 × 250 Å^3^. All models are first relaxed in an NPT ensemble (constant particle number, pressure, and temperature) for 125 ps with a timestep of 0.25 fs to eliminate the residual stress in the systems and then switched into an NVT ensemble (constant particle number, volume, and temperature) for another 125 ps. After that, the ensemble was switched to the microcanonical ensemble NVE (constant particle number, volume, and energy) for the NEMD simulations. In NEMD simulations, two ends with lengths of 0.5 nm were fixed, and another 2 nm thickness was set to apply the Langevin thermostats to build the temperature gradients. The temperature difference between the heat sink and the heat source region was 150 K. The NEMD simulations were first run for 2.5 ns to reach the steady state, and the data in the following 0.5 ns were collected to calculate thermal conductivity.

## Author Contributions

Y.Z. conceived the idea and supervised the project; Q.H. proposed the theoretical framework and did the calculations; Z.L., Y.C., and Y.Z. provided the resources; Q.H. did the experiments and conducted the material synthesis, characterization, and performance investigation; Q.H. and Y.Z. prepared the manuscript; All authors reviewed the manuscript.

## Conflicts of Interest

The authors declare no conflicts of interest.

## Supporting information




**Supporting File**: advs76841‐sup‐0001‐SuppMat.docx.

## Data Availability

The data that support the findings of this study are available from the corresponding author upon reasonable request.
