# Supplementary material for: Unveiling the Ultralow Thermal Conductivity of Hybrid Perovskites
Source: Adv Sci (Weinh). 2026 Aug 5;13(59):e76841. doi: 10.1002/advs.76841 (PMC13440369; doi:10.1002/advs.76841)
Supplement: Supplementary file 1 [file ADVS-13-e76841-s001.docx]

**Supplementary information**

**Unveiling the ultralow thermal conductivity of hybrid perovskites**

Qinqin He^1^, Yuli Chen^2^, Zhigang Li^1^ and Yanguang Zhou^[1]^*

^1^*Department of Mechanical and Aerospace Engineering, The Hong Kong University of Science and Technology, Clear Water Bay, Kowloon, Hong Kong SAR*

^2^*Institute of Solid Mechanics, Beihang University, Beijing 100191 PR China*

**Contents**

**Supplementary Note 1.** **Transducer configuration…………………………………………2**

**Supplementary Note 2.** **FDTR measurements of the sample……………………………….4**

**Supplementary Note 3.** **Thermal conductivity computational details……………………..6**

**Supplementary Note 4. Thermal conductivity contributed by the kinetic heat flux in the framework…………………………………………………………………………………...10**

**Supplementary Note 5. The validation of the calculation……………………………… 10**

**Supplementary Note 6.** **Neutron and X-ray scattering…………………………………….12**

**Supplementary Note 1. Transducer configuration**

S 1.1 The thickness of the gold layer

The sample is coated with a ~100 nm Au transducer layer, which possesses a high coefficient of thermoreflectance at the pump wavelength of 445 nm (OBIS 445-365C) and probe wavelength of 532 nm (OBIS LS532-20 )^[1]^. The thickness of the coated Au transducer is measured by a step meter by using the tape to slip a part of the Au layer (**Figure S1**).

**Figure S1**. The thickness of the gold layer measured by the step meter

S 1.2 Thermal conductivity of the gold layer

It is known that the thermal conductivity of the transducer can affect the measured results. Here, we first measure the temperature-dependent thermal conductivity of the Au layer by using the FDTR. The temperature ranges from 140 K to 380 K in increments of 30 K. In our FDTR measurements, a pump laser with an incident power of 6.23 mW and a probe laser with a wavelength of 532 nm are applied. The phase lag between the pump and probe is recorded over a modulation frequency range of 10^3^ Hz to 5×10^7^ Hz. Each position is measured at least three times to ensure the reliability of the data.

The sensitivity, which quantifies how a parameter *x* influences the measured phase (**Eq. S1**)^[2]^, is critical for obtaining reliable FDTR results.

The parameter $x$ can be any quantity of interest, e.g., the thermal conductivity *κ*, the heat capacity *C*, or the thermal boundary conductance (TBC) *G*. The high sensitivity increases the model fitting. This is because a small perturbation in $x$ yields a large change in the probe-pump phase lag, thereby enabling more robust and reliable parameter extraction. In FDTR measurements, an operational metric on sensitivity is defined, in which sensitivity is defined as the phase difference within a specified phase tolerance under the influence of a parameter $x$. For a 10% phase tolerance, the sensitivity is defined as^[3]^. This definition provides a direct measure of the influence of $x$ on the phase lag across frequency. It facilitates the identification of the frequency range used for fitting with the prescribed tolerance.

**Figure S2**. The phase lag between probe and pump laser (open blue circle) with the corresponding fitting curve (solid lines). The inset picture shows the sensitivity of thermal conductivity of the Au layer and the TBC between the Au and the glass substrate.

**Figure S2** shows the phase lag between probe and pump laser (open blue circle) and the corresponding fitting curves (solid lines). The inset of **Figure S2** shows the sensitivity of thermal conductivity of the Au layer and the TBC between the Au layer and the substrate. It is shown that the sensitivity to the thermal conductivity of the Au layer first increases and then decreases with the modulation frequency, reaching a maximum value of ~4 around 10^7^ Hz. Meanwhile, at the modulation frequency of ~10^7^ Hz, the sensitivity to TBC is only ~0.5. This indicates that the thermal penetration depth becomes comparable to the thickness of the Au layer, which therefore amplifies the transducer’s contribution to the FDTR’s phase/amplitude response at the modulation frequency of ~10^7^ Hz. At low modulation frequencies, heat penetrates more deeply, and the Au layer becomes less influential on the phase/amplitude response. **Table S1** gives the material properties used for model fitting under room temperature. Given the low sensitivity to TBC in the whole frequency range, we fix the TBC parameter when measuring the thermal conductivity of the Au layer. This is because the contribution of TBC to the FDTR response is minor and does not affect the subsequent analysis.

**Table S1**: Material properties that are needed for the sensitivity analysis of κ_Au_.

| Materials | C (10^6^ J m^-3^ K^-1^) | κ (Wm^-1^K^-1^) | d (nm) | G (W m^-2^K^-1^) |
| --- | --- | --- | --- | --- |
| Au | 2.48^*^ | TBD | 100^**^ | N.A. |
| SiO_2_ | 1.63^*^ | 1.38^*^ | Semi-infinite | 40^*^ |

*Obtained from the experiment results from Yang *et* *al* ^[4]^, **obtained from the step meter

**Supplementary Note 2. FDTR measurements of the sample**

S 2.1 The heat capacity of the sample

According to the thermal diffusion model, all related parameters should be carefully determined to ensure an accurate FDTR measurement. The heat capacity of MAPbI_3_ is measured by the differential scanning calorimetry (DSC) over 273 - 373 K. MAPbI_3_ exhibits three phases with transitions near 160 K and 320 K ^[1]^. It is known that the heat capacity at transition points is challenging to measure accurately. We also calculate the heat capacity *C_v_* using the vibrational density of states (VDOS) derived from molecular dynamics simulations

where the is the reduced Planck constant, the $n$ is the Bose-Einstein distribution. The VDOS of three phases is calculated, which then yields the temperature-dependent volumetric heat capacities across all three phases. It is noted that the measured heat capacity is slightly higher than the calculated value (**Figure S3**), which may be due to the phase transition near 320 K. In our measurements, we use the *C_v_* of the orthorhombic phase in the temperature range of < 160 K, the *C_v_* of the tetragonal phase in 170 -320 K, and the *C_v_* of the cubic phase when the temperature is above 320 K.

**Figure S3**. Volumetric heat capacities for the different phases were obtained from simulations, and experimental values were measured by DSC. The label “vdos-orth-100 K” indicates that the heat capacity curve was computed from the VDOS derived from the orthorhombic phase at 100 K and then used to evaluate heat capacity over a range of temperatures.

S 2.2 The sensitivity analysis of the FDTR test

The test settings, e.g., the radius of the laser and the thickness of the transducer, are usually optimized to obtain a high sensitivity of the measured property. This increases the reliability of the measured value. The sensitivity to the measured thermal conductivity reaches a maximum value of ~1.3 at a modulation frequency of ~1.0×10^7^ Hz (**Figure 1d** in our main text). At this modulation frequency, the thermal penetration depth becomes large enough to reach the near-surface region of the sample. **Table S2** gives the material properties used for model fitting of Au and MAPbI_3_ under room temperature. It is noted that the sensitivity of TBC between Au and MAPbI_3_ varies largely when the corresponding TBC changes from 10 Mm^-2^K^-1^ to 50 Mm^-2^K^-1^. However, the variation of the fitted κ of MAPbI_3_ is within 10% even when the TBC is changed from 10 Mm^-2^K^-1^ to 50 Mm^-2^K^-1^. Therefore, the TBC between Au and MAPbI_3_ is fixed to 50 Mm^-2^K^-1^ in all our measurements.

**Table S2**: Material properties that are needed for the model fitting of κ-MAPbI_3_ at RT.

| Materials | C (10^6^ J m^-3^ K^-1^) | κ (Wm^-1^K^-1^) | d (nm) | G (W m^-2^K^-1^) |
| --- | --- | --- | --- | --- |
| Au | 2.48^*^ | 200^**^ | 100^**^ | N.A. |
| MAPbI_3_ | 1.28^***^ | TBD | Semi-infinite | 50^****^ |

*Obtained from the experiment results from Yang *et* *al* ^[4]^, **obtained from the FDTR measurement and step meter in **SI note 1**, ***obtained from the heat capacity calculation in **SI note 2.1**, ******** TBC between Au and MAPbI_3_ is fixed to 50 Mm^-2^K^-1^ in all our measurements.

S 2.3 The fitting results of different temperatures

**Figure S4** shows the measured thermal conductivity of MAPbI_3_ under different temperatures. The mean value is calculated simply by averaging the fitting results.

**Figure S4**. The measured thermal conductivities of MAPbI_3_ under different temperatures were measured by the FDTR apparatus.

**Supplementary Note 3. Thermal conductivity calculation details**

All the thermal conductivity calculations are averaged by at least 12 independent simulations (**Figure S5**).

**Figure S5**. The thermal conductivity versus autocorrelation time of 12 independent simulations and the corresponding average value.

The calculation of the thermal conductivity contributed by potential heat flux is

$$\kappa=\frac{V}{3k_{b}T^{2}}\int_{0}^{\infty} \left\langle\vec{Q}^{pot}(\tau)\vec{Q}^{pot}(0)\right\rangle d\tau$$

And the thermal conductivity contributed from the kinetic heat flux $\vec{Q}^{kin}$ is calculated via

$$\kappa=\frac{V}{3k_{b}T^{2}}\int_{0}^{\infty} \left\langle\vec{Q}^{kin}(\tau)\vec{Q}^{kin}(0)\right\rangle d\tau$$

The thermal conductivity of the total heat flux, the potential heat flux, and the kinetic heat flux contributed by the whole system and the framework have been shown in **Figure S6**. Our results show that the thermal conductivity contributed by the kinetic heat flux is negligible. Therefore, the thermal conductivity of MAPbI_3_ can be calculated using the potential heat flux.

**Figure S6**. The thermal conductivity versus autocorrelation time contributed from total, potential, and kinetic heat flux in (a) MAPbI_3_ and (b) the framework at 300 K. The thermal conductivity of the total heat flux and the potential heat flux converge to the same value, while the thermal conductivity of the kinetic heat flux converges to nearly zero.

Unlike ions in the framework, which vibrate around their equilibrium positions, organic molecules can largely diffuse in the cage.

**Figure S7**. (a) The dynamic trajectory of organic molecules shows that the closest intermolecular distance exceeds 2.5 Å. (b) The thermal conductivity of MAPbI_3_ decomposes into the total value, conduction term, and potential term. The similarity of these three values indicates that the heat transfer within MAPbI_3_ is mainly through vibration.

**Figure S7** presents the thermal conductivity decomposition for the organic molecules, from which the diffusion term is negligible. This demonstrates that the heat transfer within organic molecules is also mainly through the propagation modes. The structure of the MAPbI_3_ provides further support for this conclusion (**Figure S7a**), since the center distance between two organic molecule clusters is around 6 Å, and the molecular dynamics trajectory shows that the closest intermolecular distance exceeds 2.5 Å. This separation is larger than the balance distance of the interaction force, indicating that direct intermolecular coupling is weak.

The vibrational spectrum of organic molecules shows that the vibrational frequency of organic molecules varies from 0 to 100 THz (**Figure S8**). We should specifically state that both results in **Figure S8** are calculated using the MYP potential, and we found that the results fit well with the *ab initio* molecular dynamics results calculated by Yue *et al*.^[5]^, which further validated the reliability of the MYP potential.

We further calculated the participation ratio (PR) to quantitatively characterize the modal localization. **Figure S8c** shows the PR of the cubic phase of MAPbI_3_. The PR of the acoustic modes at the Gamma point is 1, indicating a perfect in‑phase motion of all atoms in each unit cell. This is consistent with our spectral energy density (SED) analysis (**Figure 3a** in the Manuscript). Meanwhile, PR generally decreases with vibrational frequency, which stems from the reduced weight participation of heavy Pb and I atoms. Furthermore, the PR can be as high as 0.8 for these vibrations with frequencies lower than 5 THz. This indicates that these low-frequency vibrations are extended in MAPbI_3_, which also agrees well with our calculated SED spectrum, i.e., these low-frequency vibrations show a clear dispersion in the SED spectrum. This PR analysis further supports that the thermal energy in MAPbI_3_ is effectively transferred through vibrations.

**Figure S8**. (a) The phonon dispersion of MAPbI_3_ at the cubic phase and (b) the corresponding phonon density of states (PDOS) of organic molecules (OMs) and the framework. (c) The participation ratio of MAPbI_3_.

**Supplementary Note 4. Thermal conductivity contributed by the kinetic heat flux in the framework**

When organic molecules are fixed, no energy transfer occurs between organic molecules. As a result, the thermal energy transfer is limited in the framework of MAPbI_3_. As discussed above, the total heat flux includes both the potential and the kinetic heat flux, and the contribution to the thermal conductivity from the kinetic heat flux can be ignored (**Figure S9**).

**Figure S9**. The thermal conductivity contributed from the kinetic heat flux of the framework with different organic molecule states. (a) Organic molecules (OMs) are randomly rotated, and (b) organic molecules are orderly fixed.

**Supplementary Note 5. The validation of the calculation**

We have noted that the calculations of hybrid perovskites MAPbI_3_ and MAPbBr_3_ by Weadock et al.^[6]^ are also based on the classical MYP potential. In that reference, the calculated diffuse scattering agrees well with the measured results (**Figures 4**, **S3,** and **S16** in Ref. [6]). We further reproduce the diffuse scattering plots using the dynamic-structure-factor code ^[7]^, which agrees well with the experimental measurements from Ref. [6].

We also calculate the autocorrelation function (ACF) of the rotation of the C-N bond, which indicates the relative change of the rotation of the C-N bond. For instance, the ACF of the unity means the C-N bond rotates a little with respect to its initial position. The ACF of zero indicates the position of the C-N bond changes significantly compared to its initial position. The ACF of the C-N bond was calculated using the instantaneous C-N vector $\vec{b}_{i}^{(t)}(T)$ and the corresponding reference at *t* = 0, i.e., $\vec{b}_{i}^{(0)}(T)$.

(S5)

where *t_tot_* denotes the simulation time, and *t*_0_ is the possible initial time. Our results show that the ACF decreases quickly from unity to small values when the temperature is over 100 K. This means the C-N bond rotates largely at temperatures over 100 K (**Figure S10**), which agrees with the observations of Mattoni *et al.* ^[8]^. When the temperature is lower than 100 K (**Figure S10**), the ACF stabilizes around a high value approaching unity, which indicates the C-N bond rotates a little compared to its initial position. This also agrees with the results of Mattoni *et al.* ^[8]^. Furthermore, our calculated ACF here agrees well with the values computed using *ab initio* molecular dynamics simulations ^[9]^ (**Figure S10**), and the trend is consistent with the experimental observations^[10]^ in which the quasi-elastic neutron scattering data demonstrate that the rotation of the organic molecules increases with the rise of temperature.

**Figure S10**. RACF of organic molecules changes with the temperature and the result of *ab initio* molecular dynamics simulations ^[9]^.

We further test the influence of the size effect and the simulation time on our calculated thermal conductivity (**Figure S11**), and find our calculated results are converged. The agreement between our measured thermal conductivity and calculated values in the temperature range from ~100 K to ~300 K (**Figure 2a** in our manuscript) demonstrates the validity of the adopted MYP in this manuscript.

**Figure S11**. (a) Size effect of the EMD simulation. (b) thermal conductivity calculation under different simulation times.

Overall, based on the discussions above, we conclude that the adopted MYP in this manuscript can well capture the thermal transport properties of MAPbI_3_ and the dynamics of carriers in MAPbI_3_.

To validate the robustness of DOD metrics, we also calculated the DOD of the organic molecules in MAPbBr3. The temperature-dependent Raman spectra of MAPbBr_3_ ^[11]^ exhibit a notable broadening and eventual disappearance of the peak at 994 cm⁻¹ as temperature increases, which reflects the growing disorder of organic cations within the lattice. As shown in **Figure S12**, the DOD value rises with temperature, which is consistent with the Raman observations.

Beyond the perovskite system, we extended the DOD analysis to water molecules by calculating the rotation of each O-H bond over a temperature range of 50K to 500K. It is well known that the water molecules movement is increasing with the temperature rise. Our DOD calculations have successfully captured this trend, as shown in **Figure S12**. This result confirms that the DOD metric is not limited to MAPbI3 but can also be applied to diverse molecular systems.

**Figure S12**. The DOD of organic molecules in MAPbBr_3_ and water molecules when the temperature increases.

**Supplementary Note 6. Neutron and X-ray scattering**

For the neutron and X-ray scattering calculations, positions and velocities of all atoms in the system at various temperatures are dumped every 50 fs. The calculation was processed through the Python code developed by Sterling *et al*^[7]^.

The X-ray scattering spectra at various temperatures are shown in **Figure S13**, where scattering intensity is extracted along selected directions in the Brillouin zone. **Figure S13a** shows the X-ray intensity at the plane (-1:H:2, 0, 2.5), where the intensity at the high-symmetry points decreases with the increase of temperature, consistent with the fact that the atomic vibrational amplitudes are small at low temperatures. **Figure S13** plots the X-ray intensity at the plane (-1:H:2, 0.5, 2.5), which probes the diffuse rods and exhibits a similar temperature trend to the high-symmetry directions. In contrast, **Figure S13c** samples a region (−1:H:2, 0.25, 2.5) away from both the high-symmetry points and diffuse rods. Along this plane, the intensity decreases with the increase in temperature. This is because the signal at (−1:H:2, 0.25, 2.5) is dominated by the inelastic scattering, which becomes weak at low temperatures. Meanwhile, the X-ray intensity for MAPbI_3_ with random, randomly fixed, and orderly fixed organic molecules along K = 0.25 is also calculated (**Figure S14**). Our results show that the configuration with orderly fixed organic molecules exhibits the lowest intensity. This is because the X-ray mostly represents the vibration of the framework (heavy atoms), and the framework experiences the least inelastic scattering when the organic molecules are orderly fixed. This is consistent with the phenomenon that the system with fixed organic molecules possesses the highest thermal conductivity compared to that with random or randomly fixed organic molecules.

**Figure S13**. (a) The X-ray intensity at the plane (-1:H:2, 0, 2.5). The high-symmetric point (0, 0, 2.5) obtained an intensity decrease with temperature. (b) The X-ray intensity at the plane (-1:H:2, 0.5, 2.5), reflecting the intensity at the Brillouin zone edge. (c) The X-ray intensity at the plane (-1:H:2, 0.25, 2.5), which can properly reflect the inelastic scattering at the background intensity. The rlu stands for reciprocal lattice unit.

**Figure S14**. The X-ray intensity at the area (-1:H:2, 0.25, 2.5) for systems with random, randomly fixed, and orderly fixed organic molecules at 350 K. The area (-1:H:2, 0.25, 2.5) indicates the background of the k-space, avoiding the high-symmetry point and diffuse rods. The low intensity of the structure with orderly fixed organic molecules indicates a lower inelastic scattering.

**Figure S15**. (a-c) The neutron scattering spectra for hybrid perovskite MAPbI_3_ at 80 K, 350 K, and 500 K, respectively. (d-f) The neutron scatterings of MAPbI_3_ with random, randomly fixed, and orderly fixed organic molecules at 350 K.

As mentioned in our main text, the X-ray is sensitive to heavy atoms, and the neutron scattering is sensitive to light atoms. **Figures S15a-c** plot the neutron scattering of MAPbI_3_ at 80 K, 350 K, and 500 K. **Figures S15d-f** present the neutron scattering of the MAPbI_3_ with random, randomly fixed, and orderly fixed organic molecules at 350 K.

**Reference**

1. G. Wang, H. Fan, Z. Chen, et al., “Tuning Thermal Conductivity of Hybrid Perovskites through Halide Alloying,” *Advanced Science* *11*, no. 25 (2024): 2401194, https://doi.org/10.1002/advs.202401194.

2. A. J. Schmidt, R. Cheaito, and M. Chiesa, “A Frequency-Domain Thermoreflectance Method for the Characterization of Thermal Properties,” *Review of Scientific Instruments* *80*, no. 9 (2009): 094901, https://doi.org/10.1063/1.3212673.

3. J. Yang, C. Maragliano, and A. J. Schmidt, “Thermal Property Microscopy with Frequency Domain Thermoreflectance,” *Review of Scientific Instruments* *84*, no. 10 (2013): 104904, https://doi.org/10.1063/1.4824143.

4. J. Yang, E. Ziade, and A. J. Schmidt, “Uncertainty Analysis of Thermoreflectance Measurements,” *Review of Scientific Instruments* *87*, no. 1 (2016): 014901, https://doi.org/10.1063/1.4939671.

5. S.-Y. Yue, X. Zhang, G. Qin, J. Yang, and M. Hu, “Insight into the Collective Vibrational Modes Driving Ultralow Thermal Conductivity of Perovskite Solar Cells,” *Physical Review B* *94*, no. 11 (2016): 115427, https://doi.org/10.1103/PhysRevB.94.115427.

6. N. J. Weadock, T. C. Sterling, J. A. Vigil, et al., “The Nature of Dynamic Local Order in CH3NH3PbI3 and CH3NH3PbBr3,” *Joule* *7*, no. 5 (2023): 1051–1066, https://doi.org/10.1016/j.joule.2023.03.017.

7. T. C. Sterling, Pynamic-Structure-Factor, GitHub Repository, 2025. https://github.com/tyst3273/pynamic-structure-factor.

8. A. Mattoni, A. Filippetti, M. I. Saba, and P. Delugas, “Methylammonium Rotational Dynamics in Lead Halide Perovskite by Classical Molecular Dynamics: The Role of Temperature,” *The Journal of Physical Chemistry C* *119*, no. 30 (2015): 17421–17428, https://doi.org/10.1021/acs.jpcc.5b04283.

9. M. A. Carignano, A. Kachmar, and J. Hutter, “Thermal Effects on CH3NH3PbI3 Perovskite from Ab Initio Molecular Dynamics Simulations,” *The Journal of Physical Chemistry C* *119*, no. 17 (2015): 8991–8997, https://doi.org/10.1021/jp510568n.

10. A. M. A. Leguy, J. M. Frost, A. P. McMahon, et al., “The Dynamics of Methylammonium Ions in Hybrid Organic–Inorganic Perovskite Solar Cells,” *Nature Communications* *6*, no. 1 (2015): 7124, https://doi.org/10.1038/ncomms8124.

11. A. M. A. Leguy, A. R. Goñi, J. M. Frost, et al., “Dynamic Disorder, Phonon Lifetimes, and the Assignment of Modes to the Vibrational Spectra of Methylammonium Lead Halide Perovskites,” *Physical Chemistry Chemical Physics* *18*, no. 39 (2559): 27051–27066, https://doi.org/10.1039/C6CP03474H.

1. ^*^Author to whom all correspondence should be addressed. Email: [maeygzhou@ust.hk](mailto:maeygzhou@ust.hk) (Y. Zhou)
